# Registration Reform and the Role of Political Connections in the Initial Public Offering Process: Evidence From China

**DOI:** 10.3389/fpsyg.2022.853644

**Published:** 2022-05-03

**Authors:** Xihao Wu, Qing Zhou

**Affiliations:** ^1^School of Management, Hainan University, Haikou, China; ^2^The Research Institute of International People-to-People Exchange for Accounting, Haikou, China

**Keywords:** approval system, registration system reform, social network theory, political connections, intermediate business connections, IPO approval

## Abstract

Registration system reform is a significant and important event in the marketization of the Chinese stock market. Using social network theory, we examine how this institutional change affects the function of the network in initial public offering (IPO) activities. As an exogenous event, registration system reform provides us a good condition to conduct Different-in-Different analysis. Using 1,364 eligible samples of IPO applications in Chinese stock market from 2016 to 2021, the results show that the enterprise’s political connections (PCs) have a positive impact on IPO approval. However, registration system reform mitigates the role of PCs in IPO approval. In particular, attenuation of the efficiency of PCs under the registration system mainly exists in older firms that have long relationships with the government. The registration system can weaken the positive impact of PCs on IPO approval, even under high economic policy uncertainty where endorsements by the government are most effective. Furthermore, under the current Chinese double-track system, which includes both an approval system and a registration system, we find that enterprises with PCs are more likely to stay in the approval system to go public in the multiple application rounds, while enterprises that are not politically connected are more likely to switch the issuance system used from the approval system to the registration system. Lastly, we find that intermediate business connections, as an alternative network, can significantly improve the probability of their clients’ IPO approval, and that registration system reform has strengthened the function of intermediate business connections in IPO approval.

## Introduction

The integrate literature of behavior theory and finance has proposed that micro individual psychology and behaviors affect the development of the financial market (Mizruchi and Stearns, 2001; Hochberg et al., 2007). Financial participants, including enterprises, investors, intermediary agencies, and government regulators, enact different business psychology and decision-making behaviors under different institutional environments (Peng and Martina Quan, 2009). Registration system reform has significantly affected financial participants’ psychology and behaviors, because it aims to move the Chinese stock market from being politically oriented to being market oriented. Specifically, under the previous approval system, government intervention in initial public offering (IPO) activities has been a serious concern. China, which is well known as a “political society,” has cultivated a common practice for enterprises to establish relationships with government officers and politicians in exchange for benefits (Chen et al., 2017). For example, when issuing IPO equity, stated-owned enterprises (SOE) and private enterprises with close connections to the government have been given priority or been reserved for capital resources (Johnson et al., 2000; Sapienza, 2002; Li and Liu, 2012; Bao et al., 2016). The approval system allows the China Securities Regulatory Commission (CSRC), a ministerial-level institution directly under the State Council, to become involved and have the power to decide the success or failure of an IPO. A variety of studies have indicated that the CSRC has taken part in serious rent-seeking behaviors in IPO activities through alliances with audit firms, sponsors, Private Equity (PE) investors, Venture Capitals (VCs), and intermediates (Liu et al., 2013; Yang, 2013; Wang and Wu, 2020; Xiong and Zhao, 2021). Thus, enterprises’ behaviors are affected by the centralized approval system and the pronounced seeking of political ties.

The new registration system emphasizes market operation and weakens government intervention. One major change is that the Issuance Examination Committee (IEC) of the CSRC has been substituted by the Listing Committee established by the Securities Exchange.^1^ The Listing Committee only conducts formal checks on materials that are released by the companies seeking an IPO. It does not make substantive judgments and leaves the decision to the market participants. Therefore, the registration system is expected to weaken the function of political connections (PCs) in IPO activities. However, government officials from the CSRC continue to be present on the Listing Committee and occupy a fairly large proportion---approximately 18%. The largest proportion of members comprise accounting firms, accounting for 28%; for example, the Growth Enterprises Market (GEM). This leads to a question about the degree of government decentralization brought about by the registration system. Thus, in our study we investigate whether the Listing Committee influences IPO activities, as a substitutionary tunnel of rent-seeking. Specifically, we examine how PCs affect IPO approval and whether the registration system can exacerbate or alleviate the effectiveness of PCs on IPO approval.^2^ We apply entropy balancing (EB-DID) regression and find that the registration system significantly mitigates the impact of PCs on IPO approval.

Furthermore, the registration system may affect entrepreneurs’ behaviors in IPO activities and such institutional change can shape entrepreneurs’ use of their PCs during the process. On the one hand, entrepreneurs may seek fewer political ties but more market ties and diversify connections to adapt to the new market-oriented trend (Zhang et al., 2016). The pressure from market competition may motivate entrepreneurs to dissolve PCs and develop their internal strength and market capability (Broschak, 2004; Peng and Zhou, 2005). On the other hand, coupled with the relatively loose IPO system, there are more regulations and restrictions; for example, a strict information disclosure system and additional delisting provisions have been established. Strong ties with government can protect entrepreneurs against the competition and uncertainties that accompany institutional change (Fan et al., 2007; Claessens et al., 2008; Liu et al., 2021). Therefore, we examine which dimensions of the registration system shape entrepreneurs’ behaviors during the IPO process, and whether their behaviors exhibit inertia even after the institutional change. In particular, we investigate the behaviors of enterprises that have applied to become public several times. We find that enterprises with strong political ties are more likely to choose the Main Board under the approval system for their IPO, whereas enterprises without PCs are more pronounced in their choice of Other Boards to serve under the registration system for the IPO.

Finally, we examine whether hindering the rent-seeking activities of government triggers enterprises to find other relationships to endorse them in IPO activities. Previous literature has found that intermediates play an important role in IPO success (Pollock and Gulati, 2007; Chuluun, 2015; Rumokoy et al., 2019). Based on the structural hole theory of social network analysis, we investigate the triangular connections among the underwriter, the IEC or the Listing Committee, and the connected auditing firms^3^ (Burt, 2001). We find that enterprises can obtain benefits in the IPO process if they hire underwriters that have a large number of business transactions with the connected auditing firms. The registration system significantly enhances the positive impact of intermediate business connections on IPO approval.

In this paper, we focus on non-state enterprises from the GEM. Non-state enterprises have strong incentives to seek PCs, as they are discriminated against in accessing financial resources and are free from the protection of state-owned assets (Li et al., 2006; Zhu and Lu, 2011). They can choose the dissolution or formation of political ties flexibly, so their social network strategies rely on their own decisions made for their own benefit (Li and Yang, 2003; Broschak, 2004). Furthermore, the GEM has experienced both the approval system and the registration system. Hence, it represents a good opportunity to construct a difference-in-difference model. With regard to the endogeneity problem, our sample comprises enterprises’ IPO applications from both the GEM and the Main Board from January 2016 to July 2021. We define GEM as the treatment group and Main Board as the control group, because the registration system has been implemented in the GEM since June 2020, but has not, as yet, been applied in the Main Board. In this way, we are able to ensure that the change of PC efficiency is fully due to registration system reform.

This paper contributes to the existing literature in three aspects. First, it expands and enriches literature about the registration system. We confirm its effectiveness to mitigate government intervention, finding that the abolition of the IEC of CRSC, the handover of substantive examination to market participants, and the optional thresholds alleviate the positive impact of PC on IPO approval. This indicates that the Chinese stock market has made a great effort to move from political orientation to market orientation. Second, this paper explores the effect of macro policy on the micro corporate level. Previous literature on the approval system or the registration system has mainly focused on the operation of the stock market; few studies have involved specific corporate decisions. The Chinese stock market currently utilizes a double-track system that entails both the approval system and the registration system. We find that the double-track system gives enterprises opportunities to choose the most advantageous system in accordance with their corporate characteristics. Enterprises with PCs are more likely to remain in the approval system to go public in their multiple application rounds, whereas enterprises that are not politically connected are more likely to switch their issuance system from the approval system to the registration system. Third, this paper adds new evidence to studies that have embedded network theory in finance and accounting fields. This is the outcome of the institutional environment. With the marketization of the stock market, the effect of a politically centered network is diluted. Instead, the market-centered network becomes dominant. We find that the institutional change has changed enterprises’ behaviors in relation to establishing social networks, from seeking strong political ties to seeking weak but various market ties. We find that enterprises can improve their IPO approval rate via market ties. Specifically, the alliances of intermediates, such as underwriters and accounting firms, are probably a new concern during the IPO process, which suggests that the establishment of intermediates’ regulation may be needed.

The remainder of the paper is organized as follows. Section “Institutional Background and Literature Review” presents a brief overview of the institutional background to the Chinese IPO system and previous literature on networks in IPO activities. Section “Hypothesis Development” discusses the development of a number of hypotheses tested in this paper. Section “Samples, Variables, and Summary Statistics” describes the sample, variables, and descriptive statistics. Section “Empirical Analysis” outlines the empirical model and main results. Sections “Robustness Test” and “Heterogeneity Analysis” present the robustness tests and heterogeneity analysis. Section “Further Study” conducts further tests, section “Discussion” provides a discussion and section “Conclusion” draws conclusions.

## Institutional Background and Literature Review

### Background of the Chinese Initial Public Offering System

In this section, we summarize the progress of the Chinese IPO system. To do so, we refer to the new and old versions of “Securities Laws,” “Implementation Opinions of the Registration System on Growth Enterprises Market in Shenzhen Stock Exchange,” “The Examination and Approval rules of the Growth Enterprises Market and the Listing Rules of the Growth Enterprises Market of Shenzhen Stock Exchange.”

The Chinese IPO system has developed in three stages. The earliest was the quota system, which allocated stock issuance in accordance with local authorities’ recommendations (Liu et al., 2013; Chen, 2017). Each province would recommend several enterprises for IPO. In this way, the scope for rent-seeking was wide because the standard of the quota was not based on operating or profitability performance. The right to make a decision was held by the government. The intermediates, such as underwriters, simply had the function of helping to make the decision. Thus, the quota system is technically known as *ex ante* supervision. The second stage is the approval system. This has overcome the criticisms of the quota system to some extent. However, the decision-making rights still rest with the government. The issuers need to fully disclose the actual situation of the enterprise, and the CSRC decides whether it will obtain IPO approval (Liu et al., 2013; Bao et al., 2016). The approval system has been in use for a long time and is still applied today. Instead, with the registration system, companies are obliged to disclose information that is judged by the market and the public. Furthermore, additional regulations are attached to the relatively loose registration system. The establishment of an integrated information disclosure system and a strict delisting provision strengthens the validity of the registration system. Thus, the progress of the IPO system has been from government decision-making to government examination to government devolution.

The Chinese registration system seems to be more market oriented than the registration system used in the US. In the Chinese registration system, there is no government department involved in the IPO process, except some CSRC officials in the Listing Committee who format a review of materials and then all substantive reviews are handed over to intermediary agencies. However, under the US registration system, issuers are required to submit an IPO application to both the Securities and Exchange Commission (SEC) and their state (Karmel, 1987; Knight and Baker, 2007). The SEC focuses on information disclosure and each state performs a substantive review. This dual system of securities registration in the US is still controlled by the government with constitutional separation of power (Rao et al., 2000).

### Existing Research on Networks in Initial Public Offering Activities

Previous literature has divided PCs throughout the stock market into two types. The first is involved with political capital, which requires the capital market to protect stated-owed assets. For example, SOE have been given priority or been reserved for capital resources (Johnson et al., 2000; Sapienza, 2002; Li and Liu, 2012; Bao et al., 2016). Enterprises capitalized by investment institutions, such as private equity and venture capital investors, whose ownership is partly or wholly held by the state, are more likely to succeed in their IPO approval (Bruton et al., 2002; Naqi and Hettihewa, 2007; Liu et al., 2013; Wang and Wu, 2020). The second type of PCs arise from interpersonal relationships. For example, they can be established from a firm’s executives’ or directors’ working experience if they have formerly served in the government, or as a deputy of the People’s Congress, or the People’s Political Consultative Conference (Li et al., 2008; Bao et al., 2016). The PCs of board members prompt higher offer prices, lower underpricing, and lower fixed IPO costs (Francis et al., 2009). The first type are not likely to be influenced by the change of the IPO system, as political capital is set to protect national assets. In contrast, the second type is more flexible and may have a reaction to institutional change since the turnover of executives or directors can be easily controlled (Li and Yang, 2003; Broschak, 2004).

Besides political ties, market ties^4^ consist of connections with other market organizations and entities, such as suppliers, customers, and professional agencies. These also influence IPO outcomes (Cooney et al., 2015). For example, having a strategic alliance partnership can send out good signals and gain trust from investors (Gulati and Higgins, 2003; Pollock and Gulati, 2007; Owen-Smith et al., 2015; Park et al., 2016a,b). In addition, the underwriters’ network centrality and their experiences are relevant to the success of new ventures’ IPOs (Chuluun, 2015; Rumokoy et al., 2019). More importantly, the inter-connection of political ties and market ties may prompt a deeper impact. Chen et al. (2017) found that a politically connected underwriter increases the likelihood of clients’ IPO applications success. Rumokoy et al. (2019) found that PCs enhance the underwriters’ network centrality, which has a positive effect on underpricing and long-run post-IPO stock returns.

Another aspect of the literature is involved in the strategy management and social network fields focuses on the evolution from a political-centered network to a market-centered network with institutional changes. When market-supporting regulations are underdeveloped, enterprises are often forced to rely on strong networks to obtain benefits (Peng, 2004; Peng and Martina Quan, 2009). The increase in comprehensive legal frameworks and the decrease in the scale and scope of government intervention can transform networks from being based on strong ties to weak ties (Peng, 2003; Peng and Zhou, 2005). Furthermore, Zhang et al. (2016) found that new ventures more easily adapt their network strategies to external environment changes by establishing fewer political networks and more diverse market networks with the reform of marketization. A successful strategy of a firm is to constantly transform to adapt the institutional change (Krulicky and Horak, 2021). The main purpose of a managerial decision is to enhance the business competitiveness (Stefko et al., 2019). Earnings management, as a prevalent strategy, has high likelihood to achieve the required status of the enterprise in stock market (Durana et al., 2021). However, in the ear of modern information technology, big data-driven analysis can collecting information from various forums, that behavior of earnings management is detectable (Kovacova et al., 2020; Kovacova and Lewis, 2021). Establishing social networks becomes a alternative strategy to ensure the status acquisition in capital market (Bai et al., 2021).

## Hypothesis Development

### Can Political Connections Increase the Likelihood of Initial Public Offering Approval?

Political connections, as a precious capital, help enterprises to gain opportunities and be given priorities, especially in places where law enforcement is weak and the stock market is immature (Glaeser and Shleifer, 2003). The role of PCs is strong where there is government involvement. During the IPO review process, government officials in the CSRC are not only involved, but also have the discretion to determine the IPO’s success. In the previous approval system, the CSRC IEC held the largest percentage of voting rights and the feedback channel between the applicants and the IEC was not transparent. This gave the IEC opportunities to manipulate. The IEC itself may not screen out politically connected enterprises even though they are not qualified. Furthermore, these enterprises may not be performing well in the long term. Wang and Wu (2020) found that although enterprises with PCs were more likely to obtain IPO approval from the CSRC, they do not experience greater improvements in financial performance, corporate governance, or innovation output after receiving equity financing from the stock market. In the new registration system, although the IEC has been replaced by the Listing Committee, which is monitored by the stock exchange, CSRC government officials are still listed as members of the Listing Committee, taking the second greatest proportion (18%) of voting rights (taking the GEM as an example). Therefore, the first hypothesis was developed as follows:

**H1:** Political connections increase the likelihood of IPO approval.

### Can the Registration System Mitigate Government Intervention in the Initial Public Offering Process?

In the Chinese approval system, the government rent-seeking behaviors are severe, and the consequences of PCs are detrimental to the whole capital market due to the misallocation of financial resources (Du et al., 2013). The new registration system emphasizes the market-oriented stock market. First, the registration system abolished the IEC and established the Listing Committee, which is monitored by the stock exchange and is out of the control of government departments. This can weaken the role of administrative power in the process of IPO examination and review. Second, the competent authority (the Listing Committee) no longer performs substantive examinations. Instead, it requires a company seeking an IPO to disclose information, which will be judged by the market participants. Third, the thresholds of IPOs are various and any one qualified feature can be accepted. That is, it is difficult to refuse an IPO application by manipulating the entry standards.^5^ Therefore, the new registration system leaves almost no room for government officials and enterprises to ally together to undertake rent-seeking in IPO activities. Thus, we developed the second hypothesis:

**H2:** Registration mitigates the positive effects of PCs on IPO approval.

## Samples, Variables, and Summary Statistics

### Data Collection

We selected IPO applications with an acceptance date between January 2016 and June 2021 in the GEM and the Main Board (including the Small and Medium Enterprises’ board, which merged with the Main Board from April 2021). Please note that our sample of the IPO decisions consisted of suspension of voting or review, termination of the review or registration, and committee rejection or approval. Suspension or termination that were due to the postponement of submission of the required documents or voluntary revocation of an application were regarded as committee rejection. Such decisions imposed on firms generally involved matters of significance and equated to new declarations, in which firms were waiting to arrange resumption of the examination. We excluded the stated-owned firms as they naturally have PCs since they are under the control of the government. Financial firms were also exempted. Finally, we obtained 1,364 eligible samples of applications, of these 1,134 were approved and 230 were rejected. We manually collected the information on executives’ and directors’ PCs from the IPO prospectuses of each firm that is described in the above sample. All data were collected from the WIND database.

### Variables

The dependent variable was IPO approval, which was defined as a dummy variable APPROVAL that equaled 1 if the firm’s IPO application was approved by the IEC or the Listing Committee on the meeting date and 0 otherwise. The key independent variable was PC. Following Fan et al. (2007) and Liu et al. (2013), we defined PC as a dummy variable that equaled 1 if either a firm’s CEO, chairman, independent director or other directors formerly served in the government, or as a deputy of the People’s Congress, or the People’s Political Consultative Conference. The most important variable was Registration, which equaled 1 if applications fell under the registration system and 0 under the approval system. We also used another form to represent registration. We divided the sample into a treatment group and a control group. We set treatment as equal to 1 when the firms were in the GEM and 0 when firms were in the Main Board. We also used “Post” to differentiate between before and after the implementation of the registration system. Therefore, the interaction term Treat*Post was the same as registration. The interaction term (Regi*PC) represents the registration mitigation of the impact of PCs on IPO approval.

For control variables, we used TOP_Underwriter, TOP_Auditing, LNSIZE, LEV, Current_ratio, ROE, Margin and Growth according to previous research and the terms of issuing and listing of the GEM. Specifically, we defined Top_Underwriter and Top_Auditing as the top ten underwriters and auditing firms from the ranking list provided by the CSRC and the Chinese Institute of Certified Public Accountants. The financial data LNSIZE, LEV, Current_ratio, ROE, Margin and Growth extracted from WIND were the average figures for the latest 3 years before the year of application submission. Detailed definitions of all the variables are reported in Appendix Table A.

### Summary of Statistics

Table 1 reports the descriptive statistics of our main variables. For the dependent variable, the average approval rate is 0.831, indicating that approximately 83.1% of total IPO applications were approved in the sum sample of enterprises under both approval and registration systems. The mean of registration is 0.19, implying that the number of applications under the registration system within a year is approximately one-fifth of the number of applications under the approval system over five-and-a-half years. This achieves the anticipated goal of the introduction of the registration system, which was to give opportunities for various enterprises to go public rapidly.

**TABLE 1:** Summary statistics.

Variable	Obs.	Mean	Standard deviation	Minimum	Median	Maximum
Approval	1364	0.831	0.375	0	1	1
Registration	1364	0.190	0.392	0	0	1
PC	1364	0.378	0.485	0	0	1
PC_CEO/Chairman	1364	0.167	0.373	0	0	1
PC_Otherdirectors	1364	0.261	0.439	0	0	1
NumberofPC	1364	0.520	0.682	0	0	4
Top_underwriter	1364	0.356	0.479	0	0	1
Top_auditing	1364	0.410	0.492	0	0	1
Lnsize	1364	16.19	4.931	8.948	19.65	25.01
Lev(%)	1364	41.18	16.06	4.760	40.82	92.76
Current ratio	1364	2.364	1.874	0.214	1.830	20.72
Roe(%)	1364	21.97	9.930	–12.15	19.98	83.57
Margin(%)	1364	37.40	16.54	2.728	34.47	99.11
Growth(%)	1364	11.95	23.92	–42.71	0.365	406.3

*This table reports the summary statistics for the full sample with during January 2016–June 2021.*

With regard to the independent variables, 37.8% of our sample had PCs. This consisted of 16.7% of executives (CEO/chairman) and 26.1% of other directors. Moreover, the maximum number of PCs of any particular enterprise was four, which means that, at most, there were four politically connected executives or directors on the board. For control variables, among the IPO applications, 35.6% had hired a top underwriter and 41% hired top auditing firms. A company submitting an IPO application had, on average, RMB 107.45 billion (=e^16^.^19^/100) total assets, 41.18% leverage, 2.364 current ratio, 21.97% return on equity, a 37.4% net profit margin, and an 11.95% growth of revenue over the 3 years prior to the application.

### Univariate Test

The univariate test in Table 2 presents the differences in the IPO approvals of enterprises with and without PCs under the approval and registration systems, respectively. We used the full sample to test for differences in mean and the result shows that enterprises with PCs under the approval system exhibited significantly higher IPO approval than non-politically connected enterprises. Statistically, the average IPO approval rate of enterprises without PCs is 73.9% and that of enterprises with PCs is 89.4% under the approval system. The difference is at the 1% significance level. In contrast, the IPO approval rate of politically connected enterprises (93.2%) is less than that of enterprises without PCs (97.3%) under the registration system, although the difference is not significant. From the mean of difference-in-difference, the registration system significantly affects the impact of PCs on IPO approval.

**TABLE 2:** Univariate test.

		Obs.	Mean(%)	Diff(%)(PC-NPC)	*P*-value
Approval system	NPC	663	73.9	15.5	0.000***
	PC	442	89.4		
Registration system	NPC	186	97.3	–4.2	0.407
	PC	73	93.2		
Diff-in-Diff				–19.6	0.000***

*This table presents the differences in the IPO approvals of enterprises with and without political connections under the approval and registration systems, respectively.*

*z-Statistics in parentheses.*

***p < 0.01.

### Distribution Statistics

During the process of data collection, we found that some firms chose to go public using a different IPO system in their second or third round of IPO application. Theoretically, when the company fails to apply for an IPO, it can continue to apply until it is successful. However, if there is a difference between the approval rate for politically connected enterprises and non-politically connected enterprises under the approval system and the registration system, enterprises tend to switch to the IPO system that would be most beneficial to their IPO success.

From Table 3, we can see that only 28 reapplications switched from the approval system to the registration system, making up 2.05% of the full sample. Furthermore, politically connected enterprises accounted for 0.97% and gained 100% IPO success. Non-politically connected enterprises accounted for 2.71% and gained 91.3% IPO success. Compared to the IPO success rate of non-politically connected enterprises (73.9%) under the approval system, the IPO success rate of non-politically connected enterprises (91.3%) after switching to the registration system is higher. This indicates that enterprises with PCs are likely to remain in the approval system to gain the benefits from PCs, while non-politically connected enterprises are prone to seizing the opportunity to switch to the registration system immediately to improve their IPO approval rate.

**TABLE 3:** Distribution statistics.

	Obs.	Obs.of Switch	Proportion	IPO Success	Proportion
NPC	849	23	2.71%	21	91.3%
PC	515	5	0.97%	5	100%
total	1364	28	2.05%	26	

*This table represents firms’ choices of different IPO systems in their multiple rounds of IPO application.*

## Empirical Analysis

### Regression Model

The above two hypotheses were tested with separate equations. For hypothesis one, we used a LOGIT model to examine whether PCs boost the IPO approval rate. The regression specification is:


(1) $$L_{n}\,\frac{P\left(\mathrm{APPROVAL}=1\right)}{1-P\left(\mathrm{APPROVAL}=1\right)}$$

$$=\alpha_{0}+\alpha_{1}\text{P}\,\text{C}+\alpha_{2}\text{T}\,\text{O}\,{\text{P}}_{-}\,\text{U}\,\text{n}\,\text{d}\,\text{e}\,\text{r}\,\text{w}\,\text{r}\,\text{i}\,\text{t}\,\text{e}\,\text{r}+\alpha_{3}\text{T}\,\text{O}\,{\text{P}}_{-}\,\text{A}\,\text{u}\,\text{d}\,\text{i}\,\text{t}\,\text{i}\,\text{n}\,\text{g}$$

$$+\alpha_{4}\mathrm{LNSIZE}+\alpha_{5}\mathrm{LEV}+\alpha_{6}\mathrm{CURRENT}+\alpha_{7}\mathrm{ROE}$$

$$+\alpha_{8}\mathrm{MARGIN}+\alpha_{9}\mathrm{GROWTH}+\alpha_{10}\mathrm{INDUSTRY}$$

$$+\alpha_{11}Y\,E\,A\,R+\varepsilon$$

APPROVAL is a dummy variable that equals 1 if the firm’s IPO application is approved by the Listing Committee or the IEC, and 0 otherwise, including the suspension of voting, revocation of an application, and rejection. PCs is set as a dummy variable that equals 1 if either a firm’s CEO, chairman, independent directors, or other directors formerly served in the government, or as a deputy of the People’s Congress, or the People’s Political Consultative Conference. We also replaced PC with PC_CEO/Chairman and PC_Otherdirector to further examine whose PCs had the greatest effect. Last, but not least, we added the number of PCs to ascertain whether they were relevant. In accordance with Du et al. (2013); Yang (2013), Chen et al. (2017), and Xiong and Zhao (2021), we used the reputation of underwriters and auditing firms (Top_Underwriter/Top_Auditing), the natural log of total assets (LNSIZE), total liability/total assets (LEV), current assets/current liabilities (CURRENT), current assets/current liabilities (ROE), profit/sales (MARGIN), and growth of revenue (GROWTH) as control variables. The classification for industry was based on CSRC. The IPO application year was fixed to control for any changes.

In hypothesis two we investigated whether registration mitigated the effectiveness of PCs on IPO approval. We used the EB-DID method to examine the influence of such an exogenous event. We defined firms on the GEM as the treatment group and firms on the Main Board as the control group. In addition, we defined Post as equal to 1 if the period was after the registration system’s implementation, and 0 otherwise. The EB matching method helps re-weight a data set based on the maximum entropy scheme (Hainmueller, 2012). We set Regi (Treat*Post) as the benchmark, and the other three sets of firms (GEM firms before the registration system’s implementation, Main Board firms before the registration system’s implementation, and Main Board firms after the registration system’s implementation) to re-weight it. Table 4 provides the comparison of before and after the matching. It shows that the skewness becomes smaller after EB. Then, we used DID to perform the regression using the following equation:


(2) $$\left(L_{n}\,\frac{P\left(\mathrm{APPROVAL}=1\right)}{1-P\left(\mathrm{APPROVAL}=1\right)}\right)$$

$$=\alpha_{0}+\alpha_{1}\text{P}\,\text{C}+\alpha_{2}\text{R}\,\text{e}\,\text{g}\,\text{i}+\alpha_{3}\text{R}\,\text{e}\,\text{g}\,\text{i}*\text{P}\,\text{C}$$

$$+\alpha_{4}{\mathrm{TOP}}_{-}\,\mathrm{Underwriter}+\alpha_{5}{\mathrm{TOP}}_{-}\,\mathrm{Auditing}+\alpha_{6}\mathrm{LNSIZE}$$

$$+\alpha_{7}\mathrm{LEV}+\alpha_{8}\mathrm{CURRENT}+\alpha_{9}\mathrm{ROE}+\alpha_{10}\mathrm{MARGIN}$$

$$+\alpha_{11}\mathrm{GROWTH}+\alpha_{12}\mathrm{INDUSTRY}+\alpha_{13}\mathrm{YEAR}+\varepsilon$$

**TABLE 4:** EB result.

Before matching	Registration system (259)			Approval system (1105)		
	Mean	Variance	Skewness	Mean	Variance	Skewness
Top_Underwriter	0.3629	0.2321	0.5701	0.3538	0.2288	0.6113
Top_Auditing	0.6448	0.2299	–0.6051	0.3548	0.2291	0.6072
LnSize	11.96	8.402	2.404	17.19	22.86	–0.5918
Lev	38.67	276.70	0.1994	41.77	258.6	0.135
Currentratio	2.549	2.507	1.507	2.321	3.739	4.253
Roe	22.3	111.2	1.406	21.89	95.72	1.601
Margin	37.08	250.2	1.096	37.47	279.3	0.9986
Growth	23.59	1272	6.405	9.219	368.5	3.006

**After Matching**	**Registration system (259)**			**Approval system (1105)**		
		
	**Mean**	**Variance**	**Skewness**	**Mean**	**Variance**	**Skewness**

Top_Underwriter	0.3629	0.2321	0.5701	0.3629	0.2314	0.5701
Top_Auditing	0.6448	0.2299	–0.6051	0.6447	0.2293	–0.6048
LnSize	11.96	8.402	2.404	11.96	11.37	2.087
Lev	38.67	247.7	0.1994	38.68	225.3	0.2182
Currentratio	2.549	2.507	1.507	2.549	3.399	3.399
Roe	22.3	111.2	1.406	22.3	107	1.617
Margin	36.78	250.2	1.096	37.08	211.4	0.9246
Growth	23.59	1272	6.405	23.59	690.7	1.74

Where “Post” is an indicator variable that equals 1 if the application time is after the registration system’s implementation, and 0 otherwise. The coefficient of interaction term (Regi*PC) measures the change in the impact of the registration system on association between PCs and IPO approval.

### Results of Regressions

This section presents the empirical results of the above two hypotheses. Table 5 shows that the PCs have a positive impact on IPO approval at a 1% significance level. In column (1) the coefficient of PC is 0.789 (*t* = 4.12), suggesting that PCs increase the likelihood of obtaining IPO approval by 78.9%. Furthermore, we divided PC into two types. Column (2) shows that politically connected CEO or chairmen have a positive impact on IPO approval, but the coefficient is not significant. Column (3) shows that politically connected other directors have a positive and significant impact on IPO approval. This can be explained by the fact that politically connected other directors are usually appointed immediately before an IPO application and immediately exit from the board after IPO success. Such PCs are established for IPO purposes. Whereas most politically connected CEOs or chairmen are the founders of companies, who coincidentally have previous experience of serving for government. Columns (4), (5), and (6) take the number of PCs into consideration and reveal that the number of PC does not have a significant impact on IPO approval, except that it may have a positive and significant impact on IPO approval when a politically connected CEO or chairman is not effective in IPO approval.

**TABLE 5:** The impact of political connection on IPO approval.

Variable	IPO approval					
	(1)	(2)	(3)	(4)	(5)	(6)
PC	0.789***			1.015***		
	(4.12)			(3.56)		
PC_CEO/Chairman		0.136			–0.233	
		(0.57)			(–0.83)	
PC_Otherdirectors			1.032***			1.145***
			(4.44)			(3.96)
NumberofPC				–0.214	0.413**	–0.113
				(–1.08)	(2.53)	(–0.66)
Top_underwriter	0.007	0.004	0.021	–0.004	0.022	0.017
	(0.04)	(0.02)	(0.12)	(–0.02)	(0.13)	(0.10)
Top_auditing	–1.424***	–1.475***	–1.429***	–1.425***	–1.441***	–1.433***
	(–7.13)	(–7.44)	(–7.13)	(–7.13)	(–7.26)	(–7.14)
Lnsize	–0.001	–0.001	–0.001	–0.004	0.004	–0.003
	(–0.03)	(–0.04)	(–0.05)	(–0.15)	(0.15)	(–0.11)
Lev	0.006	0.008	0.006	0.006	0.007	0.006
	(0.75)	(1.06)	(0.71)	(0.75)	(0.93)	(0.72)
Currentratio	–0.052	–0.047	–0.062	–0.054	–0.050	–0.064
	(–0.97)	(–0.91)	(–1.14)	(–1.00)	(–0.94)	(–1.18)
ROE	0.001	–0.002	–0.000	0.001	–0.001	–0.001
	(0.09)	(–0.23)	(–0.05)	(0.09)	(–0.14)	(–0.07)
Margin	0.001	0.001	0.001	0.001	0.001	0.001
	(0.09)	(0.22)	(0.20)	(0.09)	(0.23)	(0.23)
Growth	0.004	0.004	0.003	0.004	0.004	0.003
	(0.77)	(0.91)	(0.72)	(0.76)	(0.80)	(0.71)
Constant	1.376	1.742**	1.430	1.470*	1.462*	1.500*
	(1.59)	(2.02)	(1.63)	(1.69)	(1.68)	(1.70)
Year	Y	Y	Y	Y	Y	Y
Industry	Y	Y	Y	Y	Y	Y
Observations	1,364	1,364	1,364	1,364	1,364	1,364
Adj. *R*-squared	0.2269	0.2125	0.2307	0.2279	0.2181	0.2310

*z-Statistics in parentheses.*

***p < 0.01, **p < 0.05, *p < 0.1.

Table 6 shows that the registration system significantly alleviates the positive impact of PCs on IPO approval at a 1% level. In column (1) the coefficient of Regi*PC is –2.412 (*t* = –2.98), suggesting that the registration system reduces the impact of PCs on IPO by an average of 241.2%. The coefficient of PC is 1.443 (*t* = 3.60), indicating that PCs increase the likelihood by 144.3% of passing the IEC examination under the approval system, whereas the sum coefficient of PC and Regi*PC is –0.969, clarifying that PCs decrease the probability by 96.9% of IPO approval under the registration system. Such results illustrate that the registration system has a system of strict examination for enterprises with PCs and such institutional change may excessively correct the impact of PCs on IPO approval. Column (2) shows that the registration system significantly alleviates the impact of politically connected other directors on IPO approval. Columns (3) and (4) take the effect of number of PCs into consideration and the coefficient is still negative and significant.

**TABLE 6:** The impact of Registration System on the association between political connection and IPO approval.

Variables	IPO approval			
	(1)	(2)	(3)	(4)
Regi*PC	–2.412***		–2.356***	
	(–2.98)		(–2.91)	
Regi*PC_Otherdirectors		–3.152***		–3.134***
		(–3.58)		(–3.55)
PC	1.443***		1.816***	
	(3.60)		(3.67)	
PC_Otherdirectors		1.658***		1.749***
		(3.35)		(3.18)
Regi	2.109*	2.044*	2.126*	2.033*
	(1.85)	(1.80)	(1.86)	(1.79)
NumberofPC			–0.435	–0.116
			(–1.31)	(–0.38)
Top_underwriter	0.133	0.141	0.104	0.137
	(0.42)	(0.44)	(0.33)	(0.43)
Top_auditing	0.047	0.054	0.057	0.054
	(0.14)	(0.16)	(0.17)	(0.16)
Lnsize	0.078	0.073	0.073	0.071
	(1.32)	(1.24)	(1.24)	(1.22)
Lev	–0.005	–0.003	–0.006	–0.004
	(–0.34)	(–0.22)	(–0.39)	(–0.23)
Currentratio	–0.172	–0.172	–0.183	–0.176
	(–1.47)	(–1.46)	(–1.55)	(–1.48)
ROE	0.017	0.017	0.017	0.017
	(1.07)	(1.08)	(1.05)	(1.05)
Margin	0.003	0.004	0.004	0.005
	(0.22)	(0.34)	(0.28)	(0.36)
Growth	–0.001	–0.003	–0.001	–0.003
	(–0.23)	(–0.53)	(–0.25)	(–0.53)
Constant	–0.003	0.190	0.287	0.285
	(–0.00)	(0.12)	(0.19)	(0.18)
Year	Y	Y	Y	Y
Industry	Y	Y	Y	Y
Observations	1,364	1,364	1,364	1,364
Adj. *R*-squared	0.3220	0.3252	0.3257	0.3255

*z-Statistics in parentheses.*

***p < 0.01, *p < 0.1.

## Robustness Test

### Propensity Score Matching

We performed additional tests utilizing propensity score matching (PSM) analysis. This enabled us to reduce the different selection biases among the sample applications in various dimensions. To implement PSM, we generated a robust control group of applications under the approval system and a group of applications under the registration system. We matched each application under the approval system to another application under the registration system based on one-to-three matching of nearest neighbor with replacement using several variables—LNSIZE, LEV, Current_ratio, ROE, Margin, and Growth. Table 7 presents the results of the matched treatment and control applications. It shows that the mean value of these variables is quantitatively similar and the difference between each matched group is not statistically significant after PSM. Next, we conducted a DID regression. The results in Table 8 verify that the registration system significantly reduces the impact of PCs on IPO approval.

**TABLE 7:** Propensity score matching result.

Variable	Unmatched	Mean		%Reduce	*p* > | *t* |
	Matched	Treated	Control	| Bias|	
Top_Underwriter	U	0.36	0.35	–283.7	0.783
	M	0.33	0.37		0.436
Top_Auditing	U	0.64	0.35	99.00	0.000***
	M	0.63	0.63		0.949
LnSIZE	U	11.96	17.19	96.70	0.000***
	M	12.11	11.93		0.556
LEV	U	38.68	41.77	35.20	0.005***
	M	38.74	40.75		0.165
Current Ratio	U	2.55	2.32	28.20	0.078*
	M	2.53	2.36		0.269
ROE	U	22.30	21.89	81.1	0.553
	M	22.02	21.95		0.935
Margin	U	37.08	37.47	–303.60	0.730
	M	36.49	38.09		0.224
Growth	U	23.59	9.22	87.60	0.000***
	M	20.66	22.44		0.419

*z-Statistics in paretheses.*

***p < 0.01, *p < 0.1.

**TABLE 8:** PSM-DID for robustness test.

Variables	IPO approval			
	(1)	(2)	(3)	(4)
Regi*PC	–2.335***		–2.318***	
	(–2.75)		(–2.73)	
Regi*PC_Otherdirectors		–3.464***		–3.450***
		(–3.60)		(–3.57)
PC	1.664***		2.330***	
	(4.14)		(4.55)	
PC_Otherdirectors		2.490**		2.461**
		(2.41)		(2.39)
Regi	2.446**	2.311***	2.485**	2.669***
	(2.37)	(4.11)	(2.40)	(4.28)
NumberofPC			–0.714**	–0.411
			(–2.21)	(–1.41)
Top_underwriter	0.324	0.321	0.264	0.286
	(0.96)	(0.94)	(0.77)	(0.84)
Top_auditing	–0.013	0.011	–0.012	0.013
	(–0.04)	(0.03)	(–0.04)	(0.04)
Lnsize	0.160***	0.156***	0.153***	0.152***
	(3.01)	(2.89)	(2.86)	(2.79)
Lev	0.003	0.007	0.001	0.007
	(0.17)	(0.46)	(0.07)	(0.43)
Currentratio	–0.138	–0.128	–0.147	–0.139
	(–1.09)	(–1.01)	(–1.16)	(–1.09)
ROE	0.022	0.019	0.025	0.020
	(1.22)	(1.07)	(1.34)	(1.08)
Margin	0.000	0.004	–0.001	0.003
	(0.01)	(0.24)	(–0.09)	(0.23)
Growth	0.002	0.000	0.002	–0.000
	(0.36)	(0.02)	(0.32)	(–0.05)
Constant	–1.013	–1.053	–0.438	–0.676
	(–0.70)	(–0.70)	(–0.30)	(–0.44)
Year	Y	Y	Y	Y
Industry	Y	Y	Y	Y
Observations	550	550	550	550
Adj. *R*-squared	0.3349	0.3473	0.3449	0.3513

*z-Statistics in parentheses.*

***p < 0.01, **p < 0.05.

### Elimination of Board Difference

Different boards of the stock market have different IPO thresholds and different committee members. This can trigger an endogeneity problem in that differences between boards may result in the mitigation of the effectiveness of PCs. To address this concern, we extracted data from the GEM, as it has experienced the change from the approval system to the registration system, which provided natural conditions for us to eliminate the impact of differences between boards. We defined enterprises with PCs as the treatment group and enterprises without PCs as the control group. Furthermore, we defined Post equals 1 if the period was after the registration system’s implementation, and 0 otherwise. We used EB-DID to perform the regression and the results are shown in Table 9. We can see that the coefficients of PC in columns (1) and (2) are significantly positive, which indicates that PCs promotes IPO approval in the full sample. In columns (3) and (4), the coefficients of PC are significantly positive, suggesting that PC has a positive impact on IPO approval under the approval system. Moreover, the coefficients of PC*Post are negative at a 1% significance level, indicating that the registration system has significantly mitigated the impact of PC on IPO approval. The sums of the coefficients of PC and PC*Post are –1.229 and –0.22, respectively, clarifying that PCs decrease the probability of IPO approval under the registration system. Such results illustrate that the registration system has considerably corrected the impact of PCs on IPO approval.

**TABLE 9:** Sample of GEM.

Variables	IPO approval			
	(1)	(2)	(3)	(4)
PC	0.758	1.612***	1.685***	2.420***
	(1.59)	(2.94)	(2.61)	(3.63)
Regi*PC			–2.914***	–2.640**
			(–2.75)	(–2.49)
Regi			1.312	1.528
			(0.87)	(1.05)
NumberofPC		–1.151***		–1.080***
		(–3.34)		(–2.78)
Constant	–1.059	–0.237	–2.551	–1.628
	(–0.11)	(–0.02)	(–0.22)	(–0.14)
Controls	Y	Y	Y	Y
Year	Y	Y	Y	Y
Industry	Y	Y	Y	Y
Observations	640	640	640	640
Adj. R-squared	0.3158	0.3403	0.3431	0.3630

*z-Statistics in parentheses.*

***p < 0.01, **p < 0.05.

### Effect of a Strict Committee

The examinations by the CSRC IEC were extremely strict between October 2017 and January 2019, because there were a large number of IPO applications. Consequently, the IEC had to choose the most outstanding enterprises. During this period, the IPO fail rate hit a record low. Therefore, the IPO approval rate of non-politically connected enterprises is significantly lower than that of politically connected enterprises. This may be the result of strict examinations by the IEC, and unconnected with the issuance system. In order to eliminate the interference of such strict examination, we excluded the sample of IPO applications filed within this range and then performed the regression. We used EB-DID to perform the regression and the results are shown in Table 10. The results of columns (1) and (2) show that the coefficients of PC are significantly positive, indicating that PCs promoted IPO approval in the full sample. The coefficients of PC in columns (3) and (4) are significantly positive at the level of 1%, indicating that government intervention is heavy under the approval system. The coefficients of RSI*NSOE are all significantly positive at the level of 1%, indicating that the registration system reform has greatly reduced the efficiency of PCs. The conclusion of this paper is still robust.

**TABLE 10:** Sample eliminated the effect of strict committee.

Variables	IPO approval			
	(1)	(2)	(3)	(4)
PC	1.125***	1.507***	1.815***	2.192***
	(3.08)	(3.34)	(4.59)	(4.47)
Regi*PC			–2.690***	–2.651***
			(–3.55)	(–3.47)
Regi			2.214***	2.249***
			(2.89)	(2.99)
NumberofPC		–0.441		–0.433
		(–1.46)		(–1.33)
Constant	0.418	0.633	–0.019	0.237
	(0.27)	(0.39)	(–0.01)	(0.14)
Controls	Y	Y	Y	Y
Year	Y	Y	Y	Y
Industry	Y	Y	Y	Y
Observations	1,141	1,141	1,141	1,141
Adj. R-squared	0.2404	0.2449	0.2707	0.2748

*z-Statistics in parentheses.*

***p < 0.01.

## Heterogeneity Analysis

### Economic Policy Uncertainty Heterogeneity

External or internal differences may modify the effect of institutional change on enterprises. Economic policy uncertainty determines entrepreneurs’ propensity to develop political ties. In an environment with high economic policy uncertainty, strong ties with the government can protect entrepreneurs against the competition and uncertainties that accompany institutional change. Furthermore, informing the government about an enterprise’s activities can ease government leaders’ concerns about instability brought about by high policy uncertainty and obtain their support (Zhang et al., 2016). Additionally, strong ties with the government signal that the enterprise is endorsed by government, which mitigates the concerns about high economic policy uncertainties in the eyes of third parties. Thus, in these circumstances, PC may be sufficiently strong that such institutional change cannot change the benefits of political ties. We queried whether the registration system could alleviate the impact of PC on IPO approval in situations of high economic policy uncertainty.

We obtained the Chinese monthly economic policy uncertainty index from the *Economic Policy Uncertainty* website, which follows newspaper-based methods. Specifically, it constructs a scaled frequency count of articles about policy-related economic uncertainty in the *South China Morning Post* by flagging certain terms—policy, government, Beijing, authorities, People’s Bank of China, central bank, WTO, spending, budget, interest rates, reform, deficit, tax, and regulation (Baker et al., 2016). In order to reduce the degree of heteroscedasticity and match the fluctuation level of other variables, we used the logarithm of the economic policy uncertainty index. Then, we matched it with the month of the IPO examination meeting each year and sorted the index by quartile. We performed the regression and found that the set of the first quartile had high multicollinearity. Thus, we compared the second quartile and the last quartile, which referred to low and high economic policy uncertainty, respectively.

From columns (1) and (2) of Table 11, we can see that the registration system significantly alleviates the impact of PC on IPO approval in the environment with both low and high economic policy uncertainty. The absolute value of the coefficient of Regi*PC under high economic policy uncertainty is five times that under low economic policy uncertainty, indicating that the registration system is highly effective at mitigating government intervention even in situations of high economic policy uncertainty.

**TABLE 11:** Heterogeneity analysis.

Variables	(1)	(2)	(3)	(4)
	
	Low uncertainty	High uncertainty	Old firm	Young firm
PC	3.719***	0.167	1.437**	0.822
	(4.33)	(0.13)	(2.43)	(1.27)
Regi*PC	–3.173**	–16.262***	–19.116***	0.025
	(–2.42)	(–8.82)	(–9.45)	(0.02)
Regi	3.216	18.192***	18.063***	0.618
	(1.15)	(9.95)	(11.24)	(0.56)
Top_underwriter	2.197***	–1.564**	0.125	0.641
	(3.06)	(–2.34)	(0.22)	(1.22)
Top_auditing	–0.262	–2.552*	0.009	–0.913*
	(–0.47)	(–1.88)	(0.02)	(–1.67)
Lnsize	0.041	0.159	0.178***	–0.041
	(0.41)	(1.36)	(2.65)	(–0.48)
Lev	–0.071*	–0.044	–0.006	–0.037
	(–1.89)	(–1.37)	(–0.20)	(–1.11)
Currentratio	–0.514*	–0.069	–0.272	–0.410
	(–1.74)	(–0.36)	(–0.89)	(–1.37)
ROE	0.049	0.161***	–0.007	0.058**
	(1.56)	(2.66)	(–0.16)	(2.44)
Margin	0.005	–0.152**	0.031	–0.010
	(0.22)	(–2.15)	(1.24)	(–0.54)
Growth	–0.002	0.069**	–0.001	0.002
	(–0.16)	(2.26)	(–0.11)	(0.15)
Constant	0.727	8.356*	2.338	4.345
	(0.18)	(1.83)	(0.85)	(1.61)
Year	Y	Y	Y	Y
Industry	Y	Y	Y	Y
Observations	248	169	404	260
Adj. *R*-squared	0.3673	0.5557	0.4372	0.3609
Suest Test (P > Chi2)	–12.968***		14.203***	

*z-Statistics in parentheses.*

***p < 0.01, **p < 0.05, *p < 0.1.

### Firm Age Heterogeneity

The effect of registration system reform may be modified by different types of enterprises. On the one hand, older enterprises that have experienced previous institutional change tend to maintain PCs. The early unpleasant institutional journey reinforces their tendency to maintain strong ties with officials to survive (Zhang et al., 2016). Network imprinting suggests that network structures established initially exhibit inertia and have lasting effects, even when environment conditions have changed (Marquis, 2003). On the other hand, older enterprises have survived and established various market ties over a long operating period. Reducing political ties has a small effect on their market position. Business partners may be attracted by PCs in the early stages of an enterprise. However, business partnerships are maintained by good partnership experience. PCs have little effect on a long-term business relationship, whereas, young enterprises need PCs to consolidate their market position and gain trust from other business partners. Thus, we speculated whether the registration system could alleviate the impact of PC on IPO approval in old firms or young firms.

Chinese market-oriented institutional change is divided into several stages. The initial stage began in 1978 when the Reform and Opening-up policy was implemented. The deepening stage started in 2002 when China entered the World Trade Organization. We assumed that the concept of the PC of firms is gradually weakened with deepening marketization reforms. We sorted firms’ ages by quartile and compared the first quartile and the last quartile. The time period for the establishment of companies in the first quartile was between 1958 and 2000 and the establishment time of the last quartile companies was between 2008 and 2016.

From columns (3) and (4) of Table 11, we can see that the positive impact of PC on IPO approval mainly exists in older firms. The registration system can alleviate the efficiency of PC at the 1% significance level. In terms of young firms, the effect of PC on IPO approval is not significant and the registration system has no significant impact on the association between PC and IPO approval. The results imply that the registration system is efficient at reducing government intervention in old firms, and even breaks the network imprinting of political ties.

## Further Study

### Does Institutional Change Affect Enterprises’ Choice of Different Initial Public Offering Systems?

During the process of collecting data, we found that enterprises which met the requirements of both the approval system and the registration system had difference preferences when choosing a system to go public. Thus, we examined whether the introduction of the registration system could affect enterprises’ choice of going public in different listing boards with different listing systems. In this section, we selected another sample of firms that had applied for IPO more than once. For example, one firm failed in its first IPO application, and then began a second application. This was counted as one reapplication. If the second IPO application failed, and then it started a third application, this was counted as another reapplication. The reapplications had two scenarios: an approval system application shifted to the registration system and an approval system application remained in the approval system. Therefore, we examined the companies’ bias toward their listing system choice in the second or third applications. In other words, we examined the relationship between the politically connected characteristic of firms and the propensity to choose the registration or approval system. We designed this model to further prove that firms with PCs prefer to go public under the approval system because the registration system alleviates the effectiveness of PCs. The regression model is as follows:


(3) $$L_{n}\,\frac{P\left(\mathrm{SWITCH}=1\right)}{1-P\left(\mathrm{SWITCH}=1\right)}$$

$$=\alpha_{0}+\alpha_{1}\text{P}\,\text{C}+\alpha_{2}\text{L}\,\text{N}\,\text{S}\,\text{I}\,\text{Z}\,\text{E}+\alpha_{3}\text{L}\,\text{E}\,\text{V}+\alpha_{4}\text{C}\,\text{U}\,\text{R}\,\text{R}\,\text{E}\,\text{N}\,\text{T}$$

$$+\alpha_{5}\mathrm{ROE}+\alpha_{6}\mathrm{MARGIN}+\alpha_{7}\mathrm{GROWTH}+\alpha_{8}\mathrm{INDUSTRY}$$

$$+\alpha_{9}\mathrm{YEAR}+\varepsilon$$

SWITCH is a dummy variable that equals 1 if the reapplication is from the approval system to the registration system, 0 otherwise. We assumed that firms with PCs are less likely to choose the registration system for IPO reapplications.

Table 12 presents the analysis of the propensity of enterprises with multiple rounds of application times to choose whether to use the approval system or registration system to go public. The coefficient of PC is negative and significant regardless of whether we relax the restriction on the association between the control variables and the dependent variable. Based on column (2), enterprises with PCs are twice as less likely to choose the registration system than those with such connections and are still prone to choosing the approval system to gain benefits from such relationships.

**TABLE 12:** The propensity for registration system or approval system in multiple rounds of application among politically connected firms.

Variables	SWITCH(from Approval to Registration)	
	(1)	(2)
PC	–1.996***	–2.003**
	(–2.65)	(–2.43)
Lnsize		0.074
		(0.86)
Lev		–0.018
		(–0.44)
Currentratio		0.060
		(0.21)
ROE		–0.038
		(–0.50)
Margin		0.025
		(0.76)
Growth		–0.008
		(–0.33)
Constant	1.526***	0.625
	(3.09)	(0.25)
Observations	41	41
Pseudo R-squared	0.149	0.225

*z-Statistics in parentheses.*

***p < 0.01, **p < 0.05.

### Does the Registration System Trigger Enterprises to Find Other Relationships to Endorse Them in Initial Public Offering Activities?

Previous literature has found that intermediates play an important role in IPO success (Pollock and Gulati, 2007; Chuluun, 2015; Rumokoy et al., 2019). Although the rules of the Listing Committee prohibit firms undergoing an IPO from having direct connections with the committee members,^6^ a new form of society network among intermediate agencies has come into being and plays a vital role in IPO decisions. Based on the structural hole theory of social network analysis, we investigated the indirect connections between enterprises and the connected auditing firms via underwriters (Burt, 2001). Specifically, underwriters and auditing firms have numerous business contacts from previous IPO experiences. Auditing firms obtain business opportunities from underwriters and tend to maintain a good business relationship with them. If an auditing firm is on the committee, it is reasonable to believe that it will take care of those enterprises that have hired its business partner underwriter for their IPO. Moreover, underwriters as outliers of the Listing Committee are free of supervision and, thus, are probably the main target of enterprises seeking benefits in the IPO process. Therefore, we hypothesized that such institutional change increases the impact of intermediate business connections on IPO approval.


(4) $$L_{n}\,\frac{P\left(\mathrm{APPROVAL}=1\right)}{1-P\left(\mathrm{APPROVAL}=1\right)}$$

$$=\alpha_{0}+\alpha_{1}\text{I}\,\text{B}\,\text{C}+\alpha_{2}\text{R}\,\text{E}\,\text{G}\,\text{I}+\alpha_{3}\text{I}\,\text{B}\,\text{C}*\text{R}\,\text{E}\,\text{G}\,\text{I}$$

$$+\alpha_{4}{\mathrm{TOP}}_{-}\,\mathrm{Underwriter}+\alpha_{5}{\mathrm{TOP}}_{-}\,\mathrm{Auditing}+\alpha_{6}\mathrm{LNSIZE}$$

$$+\alpha_{7}\mathrm{LEV}+\alpha_{8}\mathrm{CURRENT}+\alpha_{9}\mathrm{ROE}+\alpha_{10}\mathrm{MARGIN}$$

$$+\alpha_{11}\mathrm{GROWTH}+\alpha_{12}\mathrm{INDUSTRY}+\alpha_{13}\mathrm{YEAR}+\varepsilon$$

The dependent variable IBC is a dummy variable which equals 1 if the underwriter employed had business contact with connected auditing firms within 4 years prior to the IPO application. The connected auditing firms refer to the firms with a representative member in the IEC or the Listing Committee in a given application year.

From Table 13, we can see that the coefficient of IBC (0.465*) is positive at 10% significance, indicating that a connected intermediate increases the IPO approval. Meanwhile, the number of intermediate business connections has no significant impact on IPO approval. From Table 13, the interaction term of Regi*IBC is positive at the 1% significance level, suggesting that the registration system strengthens the positive impact of intermediate business connections on IPO approval. Additionally, the coefficient of IBC (–0.011) shows that the causal relationship between IBC and IPO approval is negative but not significant under the approval system. In contrast, the sum coefficient of IBC and Regi*IBC (2.479) is positive under the registration system. Overall, these three coefficients explain that the registration system significantly reverses the impact of intermediate business connections on IPO approval from negative to positive. This finding proves that institutional change prompts enterprises to find alternative ways to confront uncertainty. With the introduction of the registration system, the regulation of intermediates should be established. In column (2) of Table 11, we added the number of intermediate business contacts as an additional control variable and the result also supports our findings.

**TABLE 13:** The impact of the registration system on the association between intermediate connection and IPO approval.

Variables	IPO approval			
	(1)	(2)	(3)	(4)
IBC	0.465*	0.461*	–0.011	0.381
	(1.92)	(1.68)	(–0.02)	(0.71)
Regi*IBC			2.490***	3.230***
			(2.58)	(3.03)
Regi			–0.815	–1.071
			(–0.65)	(–0.84)
NumberofIBC		0.002		–0.198**
		(0.03)		(–1.98)
Top_underwriter	–0.065	–0.067	0.113	0.389
	(–0.36)	(–0.35)	(0.35)	(1.12)
Top_auditing	–1.509***	–1.509***	–0.111	–0.102
	(–7.57)	(–7.57)	(–0.34)	(–0.31)
Lnsize	0.002	0.002	0.069	0.069
	(0.10)	(0.10)	(1.19)	(1.19)
Lev	0.007	0.007	–0.007	–0.008
	(0.97)	(0.96)	(–0.47)	(–0.49)
Currentratio	–0.051	–0.051	–0.219*	–0.227*
	(–0.97)	(–0.97)	(–1.77)	(–1.79)
ROE	–0.003	–0.003	0.018	0.016
	(–0.28)	(–0.28)	(1.08)	(1.01)
Margin	0.002	0.002	0.008	0.008
	(0.25)	(0.26)	(0.59)	(0.61)
Growth	0.004	0.004	–0.001	–0.000
	(0.87)	(0.87)	(–0.12)	(–0.06)
Constant	1.521*	1.520*	0.815	1.117
	(1.74)	(1.74)	(0.51)	(0.70)
Year	Y	Y	Y	Y
Industry	Y	Y	Y	Y
Observations	1,364	1,364	1,364	1,364
Adj. *R*-squared	0.2152	0.2152	0.3013	0.3103

*z-Statistics in parentheses.*

***p < 0.01, **p < 0.05, *p < 0.1.

## Discussion

Our main empirical results indicate that political-connected executives and directors have influence on IPO firms’ activities via increasing the likelihood of IPO approval by 78.9%. Specifically, politically connected CEO or chairmen have a positive but not significant impact on IPO approval, whereas politically connected other directors have a positive and significant impact on IPO approval. The results suggest that politically connected other directors are for IPO purpose because they are mobile, and in fact, they are immediately appointed before an IPO application and exit from the board after IPO success. By contrast, most politically connected CEOs or chairmen are the founders of companies and randomly have previous experience of serving for government. Most importantly, empirical results show that the registration system can moderate the positive association between PCs and IPO approval, significantly reducing it by an average of 241.2%. Such results illustrate that the registration system is particularly strict for enterprises with PCs and such institutional change may excessively correct the impact of PCs on IPO approval. This assumption is verified by our further study. The empirical results of further study suggest that enterprises with PCs are 199.6% less likely to choose the registration system and consistently select the approval system to go public. Last but not least, the empirical results indicate the registration system significantly reverses the impact of intermediate business connections on IPO approval from negative to positive. In sum, the above results show the effectiveness of the registration system on the mitigation of government intervene in IPO activities and enterprises have proactively developed diverse market ties to confront uncertainty with institutional changes.

The study demonstrates that registration system alleviate the effectiveness of PC and strengthen the function of intermediates business connection on IPO approval in Chinese stock market. Various types of social ties have different impacts on IPO activities. Such influences are also found in U.S. stock markets. For example, Gulati and Higgins (2003) examine a sample of U.S. biotechnology firms, and find that new issuers endorsed by prestigious Venture Capitals (VCs) are more likely to gain IPO success in the cold equity markets, whereas establishing partnership with prestigious underwriters assists new issuers’ IPO success in the hot equity markets. Chuluun (2015) investigates public equity securities in the U.S. stock market, and shows that underwriter centrality is positively associated with an offer price revision, IPO underpricing and short-term stock returns. Cooney et al. (2015) employ a sample of U.S. firms and assert that the social connection between a investment bank and an IPO firm play a significant role in determining an IPO firm’s underwriting syndicate. Owen-Smith et al. (2015) focus on U.S. firms based VCs funded in high-technology industries and propose that information and resources via VCs syndicate and director interlock networks decrease the likelihood of IPO withdrawal. Our study implies that in the phrase of Chinese institutional transition, entrepreneurs have proactively developed diverse social ties from political-orientation to market-orientation.

The presented study has limitations and we implicate several directions of future research. The findings are based on a short period after the registration system implementation. It is generally known that policy reform is far efficient at the very beginning of the implementation due to the focus of the public. We recommend that the long-term effect of the registration system reform on the government intervene during IPO process should be further examined in the future. Additionally, we only studied the influence of the registration system in the initial application stage of IPO. We leave a quite large space for future research to examine how does the registration system affect the subsequent stage of IPO, such as IPO pricing, IPO financing cost, short-term and long-term IPO performance, etc.

## Conclusion

Prior studies have examined the effects of different networks on the process of IPO under a single system. For example, a number of papers in the literature have studied PCs, underwriters’ syndication, and inter-organizations’ alliance in the approval system. The Chinese equity market gave us an opportunity to investigate how the effectiveness of a certain network of IPO activities has changed in a double-track system with the existence of both the approval system and the registration system. We found that the effectiveness of PCs—dominant under the Chinese approval system—was significantly mitigated after the introduction of the registration system. In particular, the efficiency of PC attenuated under the registration system mainly existed in old firms that have long relationships with government. The registration system can weaken the positive impact of PCs on IPO approval, even under conditions of high economic policy uncertainty where endorsements by government are most effective.

Furthermore, we examined how an institutional change shapes entrepreneurs’ use of their PCs during the process. Specifically, we found that enterprises with PCs are more likely to remain in the approval system for their public launch in multiple application rounds, whereas enterprises without PCs switch to the registration system to go public. Finally, we found an alternative network that enhanced its roles after PCs became invalid. The market ties between underwriters and connected auditing firms significantly improve the underwriters’ clients’ IPO approval rate. Further studies could focus on the method and the consequence of benefits’ exchange between underwriters and auditing firms. Our finding implies that there is an obvious trend that the Chinese stock market is moving from being politically oriented to market oriented. Enterprises’ behaviors shaped by such institutional changes have forced them to dissolve strong PCs when the costs of their maintenance exceed the benefits of IPO success. Hence, they seek diverse marketable connections. In addition, the regulation of intermediates should be integrated since it might be another method of rent-seeking.

## Data Availability Statement

The original contributions presented in the study are included in the article/supplementary material, further inquiries can be directed to the corresponding author/s.

## Author Contributions

XW was contributed to responsible for providing overall thinking. QZ was contributed to responsible for literature arrangement, model design, and data collection and analysis. Both authors contributed to the article and approved the submitted version.

## Conflict of Interest

The authors declare that the research was conducted in the absence of any commercial or financial relationships that could be construed as a potential conflict of interest.

## Publisher’s Note

All claims expressed in this article are solely those of the authors and do not necessarily represent those of their affiliated organizations, or those of the publisher, the editors and the reviewers. Any product that may be evaluated in this article, or claim that may be made by its manufacturer, is not guaranteed or endorsed by the publisher.

## Footnotes

1 The Listing Committee under the Securities and Exchange in China is similar to the United States Securities and Exchange Commission.

2 Some scholars have proposed that the registration system has opened the gates of the IPO market with a low threshold. They have stated that the registration system clearly improves the IPO approval rate irrespective of the factors involved. However, we mainly focus on the function of political connections and examine whether the Chinese stock market is still politically oriented.

3 The term “connected auditing” firms refers to the firms with a representative member in the IEC or the Listing Committee in a given application year.

4 This is known as business ties in other papers.

5 The equity resources are not infinite. If a politically connected but unqualified enterprise had obtained IPO success, a non-politically connected but qualified enterprise may have been refused. This scenario is likely to happen, as non-politically connected enterprises “give way” to politically connected ones.

6 For example, auditing firms on the committee cannot examine their IPO clients directly.

## Appendix

**APPENDIX TABLE A:** Variable definitions.

Variables	Definition
IPO Approval	Equals 1 if the firm’s IPO application is approved by the IEC or the Listing Committee on the meeting date and 0 otherwise.
PC	Equals 1 if either a firm’s CEO, chairman, independent director, or other directors formerly served in the government, or as a deputy of the People’s Congress, or the People’s Political Consultative Conference.
PC_CEO/Chairman	Equals 1 if either a firm’s CEO or chairman formerly served in the government, or as a deputy of the People’s Congress, or the People’s Political Consultative Conference.
PC_Otherdirector	Equals 1 if either a firm’s independent director or other directors formerly served in the government, or as a deputy of the People’s Congress, or the People’s Political Consultative Conference.
NumberofPC	The number of political connections the board have, including the firm’s CEO, chairman, independent director, and other directors.
Registration	Equals 1 if application is under the registration system and 0 under approval system.
TOP_Underwriter	Equals 1 if it ranks within top 10 underwriters according to market share from WIND.
TOP_Auditing	Equals 1 if it ranks within top 10 auditing/accounting firms according to the Chinese Institute of Certified Public Accountants.
LNSIZE	ln (total assets), average value of the latest 3 years before the application submission year.
LEV	Total liabilities/total assets, average value of the latest 3 years before the application submission year.
Current_ratio	Current assets/current liabilities, average value of the latest 3 years before the application submission year.
ROE	Net income/shareholders’ equity, average value of the latest 3 years before the application submission year.
Margin	Profit/sales, average value of the latest 3 years before the application submission year.
Growth	Growth of revenue, average value of the latest 3 years before the application submission year.
